# Association between fatigue, pain, digestive problems, and sleep disturbances and individuals’ health-related quality of life: a nationwide survey in South Korea

**DOI:** 10.1186/s12955-020-01408-x

**Published:** 2020-05-27

**Authors:** Younghwa Baek, Kyungsik Jung, Hoseok Kim, Siwoo Lee

**Affiliations:** Future Medicine Division, Korea Institute of Oriental Medicine, 1672 Yuseong-daero, Yuseong-gu, Daejeon, 34054 Republic of Korea

**Keywords:** Fatigue, Pain, Digestion, Sleep, Symptoms, Health-related quality of life

## Abstract

**Background:**

Physical symptoms such as fatigue, pain, digestive problems, and sleep disturbances are chief reasons individuals seek primary care, as they affect health-related quality of life. We investigated the associations between various combinations of these common symptoms and individuals’ health-related quality of life.

**Methods:**

This large-scale survey study of 1100 Koreans aged ≥19 years was conducted in 2017 using multi-stage stratified sampling based on region, sex, and age. Data were collected using questionnaires administered face-to-face; then, a linear regression analysis was performed to assess how the symptoms were related to participants’ health-related quality of life. Complex symptoms were defined as co-occurrence of two or more of the four symptoms—fatigue, pain, digestive problems, and sleep disturbances.

**Results:**

The most frequently observed stand-alone symptom was fatigue, while the most common combination was fatigue and pain. When examined individually, fatigue, digestive problems, and sleep disturbances were closely associated with mental health-related quality of life, and pain was associated with physical health-related quality of life. Complex symptoms were also related to health-related quality of life. Lower physical health-related quality of life was strongly associated when fatigue and pain or all four symptoms were co-occurring, and the lowest mental health-related quality of life was seen when all four symptoms were present, after adjusting for all variables.

**Conclusions:**

Symptoms can be present in various combinations and are significantly associated with health-related quality of life. Extra attention should be given to patterns accompanying fatigue and pain and to those involving more symptoms. This elucidated the characteristics of symptoms that affect the health-related quality of life of South Korean adults.

## Introduction

Health-related quality of life (HRQOL)—how individuals subjectively view their health and its effects on their well-being—is a key outcome when measuring one’s health status and the effects of medical treatment [1]. HRQOL is related to demographic characteristics such as sex, age, region [2–4], lifestyle factors [5, 6], emotional disorders [7], chronic conditions/diseases [8], and physical discomfort [9–11].

The most commonly reported symptoms of physical discomfort are fatigue, pain, digestive problems, and sleep disturbances [11, 12], and physical symptoms are a chief reason people seek primary care [13–15]. Moreover, as these symptoms are closely linked to each other, they tend to occur concurrently [16]. For example, fatigue is associated with sleep disorders and depression [17, 18], and pain is associated with fatigue, depressed mood, and digestive problems such as appetite loss and constipation [19]. Further, insomnia is often accompanied by various medical problems, and pain and gastrointestinal problems occur at a 25–30% higher rate in people with insomnia than in those without insomnia [20]. Consequently, multiple concurrent symptoms, defined as complex symptoms (the co-occurrence of two or more of the four commonly reported symptoms above) in the present study, are much more complex than single symptoms, and they have a strong/negative synergic influence on the progression and outcomes of medical conditions [16] as well as on HRQOL [9, 21].

The results of previous studies concerning the relationships between multiple concurrent symptoms and decreased HRQOL suggest the need for a multidisciplinary, evidence-based strategy for intervention and management. However, these studies have mainly investigated symptom clusters in individuals with a specific disease, such as cancer or multiple sclerosis [22–24]. Moreover, fatigue, pain, digestive problems, and sleep disturbances occur in a repetitive and nonspecific way, and the organic causes of such symptoms have not been clearly identified among the South Korean general population, making it difficult for medical professionals to explain them decisively [14].

This study examined nationwide data collected from the general population, considering sex, age, and region of Korea to determine how fatigue, pain, digestive problems, and sleep disturbances are related to individuals’ HRQOL. We also considered the associations between complex symptoms and individuals’ HRQOL.

## Methods

### Study design and participants

This large-scale, national survey targeted the adult general population (aged ≥19 years) from all regions in South Korea between November and December 2017. This study used a multi-stage stratified sampling method that stratifies region, sex, and age in proportion to the South Korean adult population of 2017 [25]. It first divides South Korea into five areas (Capital district, Kangwon, Chungcheong, Jeolla, and Gyeongsang) based on the 16 administrative districts. Then, sex (men and women) and age (≤ 29, 30–39, 40–49, 50–59, and ≥ 60 years) were stratified in each area. With an equation that estimates the population proportion, we calculated that we needed 1100 participants to effectively represent the stratified sampling (sampling error ± 3.0 and 95% confidence interval (CI)). The 1100 participants were distributed according to the sample size assigned to each stratum by multi-stage stratified sampling method. All participants were chosen at random in predefined survey places within the administrative districts. The inclusion criteria for participants included being a South Korean citizen aged ≥19 years, the legal age of adulthood in Korea, and providing consent to participate. Data were collected by face-to-face interviews in Korean in coordination with a social survey agency (Gallup Korea), using a structured questionnaire including questions on symptoms, HRQOL, and sociodemographic variables. Interviewers were trained and supervised according to a standardized procedure to reduce the likelihood of errors in data collection. This study was approved by an appropriate institutional review board (no. I-1708/007–003-02) and written informed consent was obtained from all participants.

### Data and measures

#### Physical symptoms

Participants’ fatigue, pain, digestive problems, sleep disturbances, symptom severity, symptom duration, and recovery level after rest were examined using a structured questionnaire comprising 12 items with a 7-point scale for responses [26, 27], which was also utilized in previous research [11, 12]. We developed a questionnaire for this study using the physical symptom items from the symptom questionnaire developed by Lee and colleagues to assess health status based on discomfort symptoms [27]. The original questionnaire is reliable (total: Cronbach’s α = 0.88, correlation coefficient = 0.631 via test-retest; four physical symptoms: Cronbach’s α = 0.87, correlation coefficient range = 0.514–0.673 via test-retest) [27] and valid (71% agreement and 0.418 kappa value, compared to a professional’s examination) [26]. The Cronbach’s alpha in this study was 0.875 (individual symptoms had Cronbach’s α αs between 0.774 and 0.865). Concerning severity of symptoms, we asked, “How severe has your fatigue been in the past 30 days?” Participants responded from 1 (*very weak*) to 7 (*very severe*). Concerning symptom duration, we asked, “For how many days has your fatigue lasted in the past 30 days?” Participants responded from 1 (*one day or less*) to 7 (*seven days or longer*). Concerning recovery, we asked, “How has your fatigue changed after resting in the past 30 days?” Participants responded from 1 (*very improved*) to 7 (*not improved at all*). Other variables were assessed in a similar fashion. Each physical symptom referred to common symptoms in people’s daily lives, and detailed examples were explained for additional clarity.

**Fig. 1:**
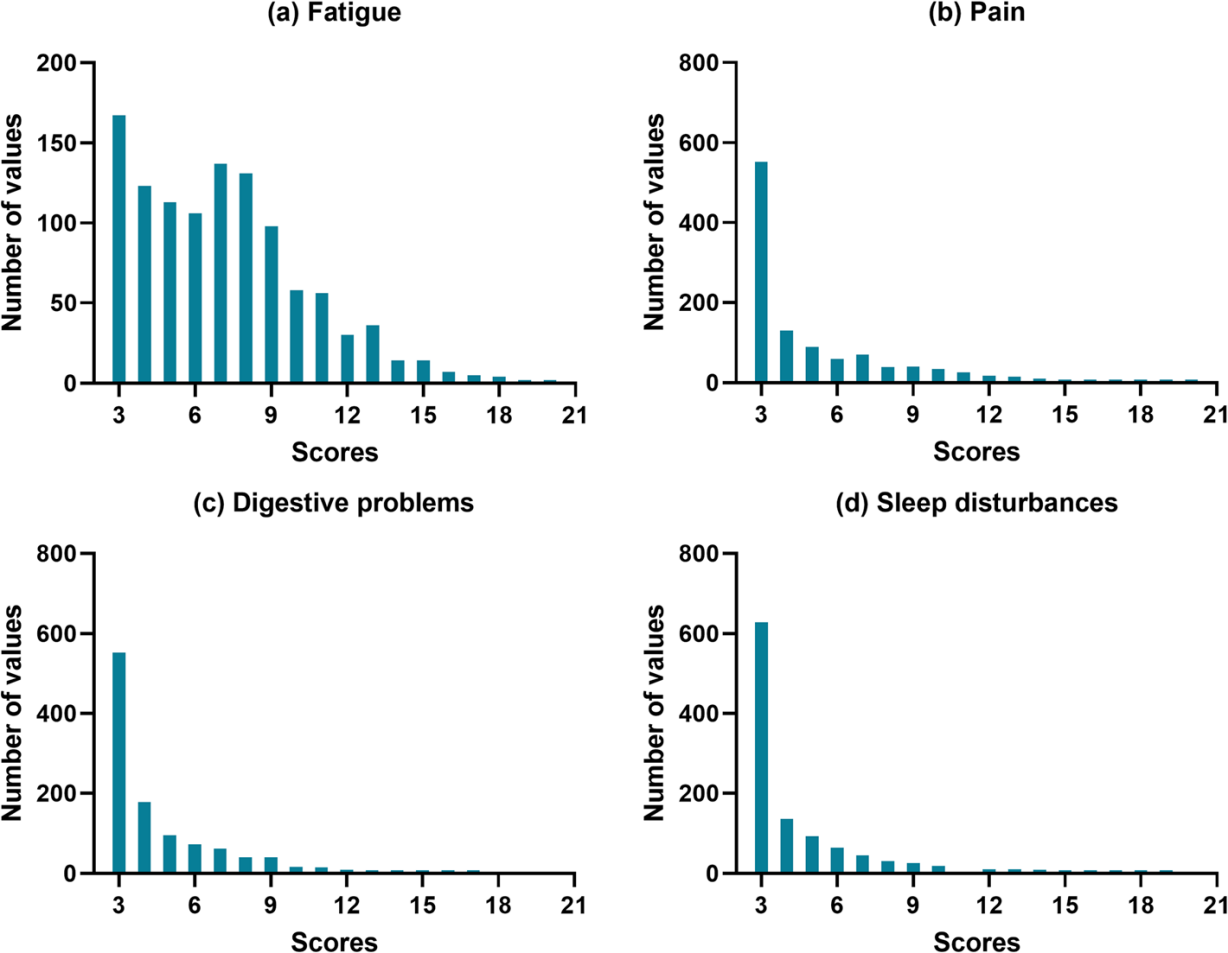
Distributions of values for each symptom

The scores of each symptom were summed (each ranged 3–21 points), and higher scores indicated greater symptom severity. The questionnaire used in this study lacks the cut-off criterion that determines the presence of each symptom because the original tool just suggested cut-off standards based on the total score of overall symptoms and there were no individual cut-off standards [27]. As shown in Fig. 1, there were differences in the distribution of scores across the individual symptoms; therefore, considering the distribution of each symptom, we established an operational definition for the presence of each symptom with a cut-off value of 7, which is the lower third of the maximum total points [21] available for each symptom. Based on the total score for each symptom, individual symptoms were interpreted as either “not present” (total score < 7) or “present” (total score ≥ 7). Regarding the number of symptoms, responses indicating the presence of fatigue, pain, digestive problems, and sleep disturbances were counted and categorized as 0, 1, 2, and ≥ 3, with higher scores indicating the presence of more symptoms. To assess the combined associations of the four symptoms, a composite symptom variable was created with 16 mutually exclusive categories. Out of the 16 symptom patterns, seven symptom patterns (*n* = 73) were excluded, and the remaining nine patterns were analyzed. This is because the frequency of the seven symptom patterns was too low (each frequency of the seven patterns ranged between 2 and 22).

#### HRQOL

HRQOL was assessed using the Optum™ Short-Form 12-Item Health Survey (SF-12 v2) [28]. The SF-12 comprised 12 items across eight domains: physical functioning, role physical, role emotional, bodily pain, general health, vitality, social functioning, and general mental health. In turn, these eight domains form two higher-order factors: a physical component summary (PCS) and a mental component summary (MCS) [28]. The SF-12 is frequently used to assess HRQOL in relation to health, and its reliability and validity have been confirmed with a wide range of populations [29]. Cronbach’s alpha for SF-12 in this study was 0.745 (PCS Cronbach’s α = 0.55, MCS Cronbach’s α = 0.66). The PCS and MCS scores ranged from 0 to 100 points, with higher scores indicating better health status [30].

#### Covariants

Sociodemographic variables, including sex, age, region, occupation, income, education, marital status, smoking, alcohol consumption, physical activity, and disease history were controlled as covariants. These variables were shown to be related to HRQOL in a previous study [2]; for example, older age, women, and rural area were associated with poor HRQOL [2–4]. Based on the distribution of the data, variables were stratified as follows (see Table 1). Occupation was classified as office job, service job, and others (including student, housewife, retired, and unemployed). Income refers to the total monthly income of a household and has four categories (less than 3000,000 South Korean Won (KRW), 3000,000–3999,999 KRW, 4000,000–4999,999 KRW, and more than 5000,000 KRW). Education was classified as below high school and above college levels based on the survey that had the following categories: below elementary school, middle school, high school, and above college. Marital status was classified as married and other, which included single, separated, and widowed. Current smokers were defined as those who had smoked more than 100 tobacco cigarettes in his or her lifetime and who currently smoked, in accordance with the standard of National Health Interview Survey [31]; all other participants were classified as nonsmokers. Current drinkers were defined as those who had consumed alcohol at least once a month for the past year; all other participants were classified as nondrinkers. Physical activity was determined according to the Korean version of the Global Physical Activity Questionnaire; participants were placed into high, moderate, or low activity groups based on previous studies [32, 33]. Regarding disease history, the presence or absence of a diagnosis of hypertension, diabetes, hyperlipidemia, cardiovascular disease, or cancer was determined in an interview by asking the following question and then listing the relevant diseases: “Have you ever been diagnosed with the following diseases by a physician?”

**Table 1:** Participants’ general characteristics

Characteristic	Men (***n*** = 545)	Women (***n*** = 555)	Total (***N*** = 1100)
Age (years)			
≤ 29	101 (18.5)	92 (16.6)	193 (17.5)
30–39	98 (18.0)	95 (17.1)	193 (17.5)
40–49	114 (20.9)	112 (20.2)	226 (20.5)
50–59	112 (20.6)	107 (19.3)	219 (19.9)
≥ 60	120 (22.0)	149 (26.8)	269 (24.5)
Mean ± SD	46.4 ± 15.5	47.9 ± 16.0	47.2 ± 15.8
Region			
Capital district	276 (50.6)	276 (49.7)	552 (50.2)
Kangwon	16 (2.9)	18 (3.2)	34 (3.1)
Chungcheong	57 (10.5)	59 (10.6)	116 (10.5)
Jeolla	54 (9.9)	58 (10.5)	112 (10.2)
Gyeongsang	142 (26.1)	144 (25.9)	286 (26.0)
Occupation			
Office	137 (25.1)	103 (18.6)	240 (21.8)
Service	340 (62.4)	223 (40.2)	563 (51.2)
Other^a^	68 (12.5)	229 (41.3)	297 (27.0)
Household income^b^ (USD)			
< 300 (< 3000)	132 (24.2)	146 (26.3)	278 (25.3)
300–399 (3000–3999)	140 (25.7)	138 (24.9)	278 (25.3)
400–499 (4000–4999)	114 (20.9)	118 (21.3)	232 (21.1)
≥ 500 (5000)	159 (29.2)	153 (27.6)	312 (28.4)
Education (over college)	277 (50.8)	199 (35.9)	476 (43.3)
Marital status (married)	361 (66.2)	391 (70.5)	752 (68.4)
Smoking (current smoker)	268 (49.2)	14 (2.5)	282 (25.6)
Alcohol consumption (current drinker)	485 (89.0)	309 (55.7)	794 (72.2)
Physical activity (high)	136 (25.0)	100 (18.0)	236 (21.5)
Disease history (yes)^c^	174 (31.9)	146 (26.3)	320 (29.1)
Symptom (3–21)^d^			
Fatigue	7.0 ± 3.1	7.2 ± 3.4	7.1 ± 3.3
Pain	4.8 ± 2.7	5.5 ± 3.5	5.1 ± 3.2
Digestive problems	4.4 ± 2.2	4.9 ± 2.7	4.6 ± 2.5
Sleep disturbances	4.3 ± 2.4	4.9 ± 3.1	4.6 ± 2.8
HRQOL (0–100)^e^			
Physical (PCS)	53.4 ± 5.2	51.4 ± 6.9	52.4 ± 6.2
Mental (MCS)	52.7 ± 6.4	52.6 ± 6.7	52.6 ± 6.5

Data are presented as n (%) or mean ± standard deviation. *HRQOL* health-related quality of life, *PCS* SF-12 Physical Component Summary, *MCS* SF-12 Mental Component Summary, *SD* standard deviation, *USD* United States dollars. ^a^Including student, housewife, retired and unemployed. ^b^unit: 10,000 South Korean won. ^**c**^Disease history comprised one or more of the following: hypertension, diabetes, hyperlipidemia, cardiovascular disease, cerebrovascular disease, or cancer. ^d^Score range of each symptoms, and higher scores were interpreted as experiencing more symptom(s). ^e^Score range of PCS and MCS; higher scores indicated better quality of life

### Statistical analyses

General characteristics, means, and standard deviations (SD) were computed for continuous variables, and numbers and percentages were computed for categorical variables. An analysis of covariance was performed to compare HRQOL scores for according to the presence of symptoms after adjusting for sex, age, and remaining three symptoms that were not being used as the dependent variable. The associations between symptoms, such as number of symptoms and individual and combined symptoms and HRQOL, were summarized with B coefficients and their 95% CIs by a linear regression analysis. Statistical analyses were conducted after controlling for sex, age, region, and other variables, such as occupation, income, education, marital status, smoking, alcohol consumption, physical activity, and disease history. Statistical analyses were performed using SPSS 24.0 (IBM Corp., Armonk, NY, USA), and the *p*-value < 0.05 was deemed significant.

## Results

### General characteristics

Participants’ general characteristics are shown in Table 1. Of the 1100 participants, 49.5% were men and 50.5% were women. Their mean age was 47.2 (SD = 15.8) years.

### HRQOL according to individual symptoms

**Fig. 2:**
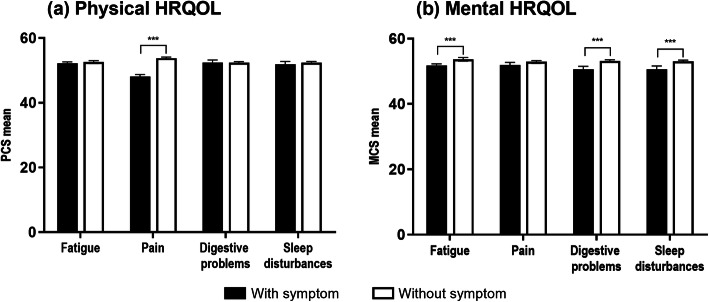
Differences in health-related quality of life per symptom type. HRQOL: health-related quality of life, PCS: SF-12 Physical Component Summary, MCS: SF-12 Mental Component Summary. ^***^P < 0.001. Analysis of covariance after adjusting for sex, age, and the remaining three symptoms not being used as the dependent variable. Bars: black indicates the presence and white indicates the absence of each symptom. (**a**) The significant mean scores of PCS were 48.1 for pain and 53.8 for no pain. (**b**) The significant mean scores of MCS were 51.8 and 53.6 for with and without fatigue, respectively; 50.6 and 53.1 for with and without digestive problems, respectively; and 50.6 and 53.0 for with and without sleep disturbances, respectively

The associations between categories of individual symptoms and HRQOL are shown in Fig. 2. Pain showed a significant difference regarding physical HRQOL, and fatigue, digestive problems, and sleep disturbances showed a significant difference regarding mental HRQOL, after adjusting for variables.

### Associations between number of symptoms and HRQOL

**Fig. 3:**
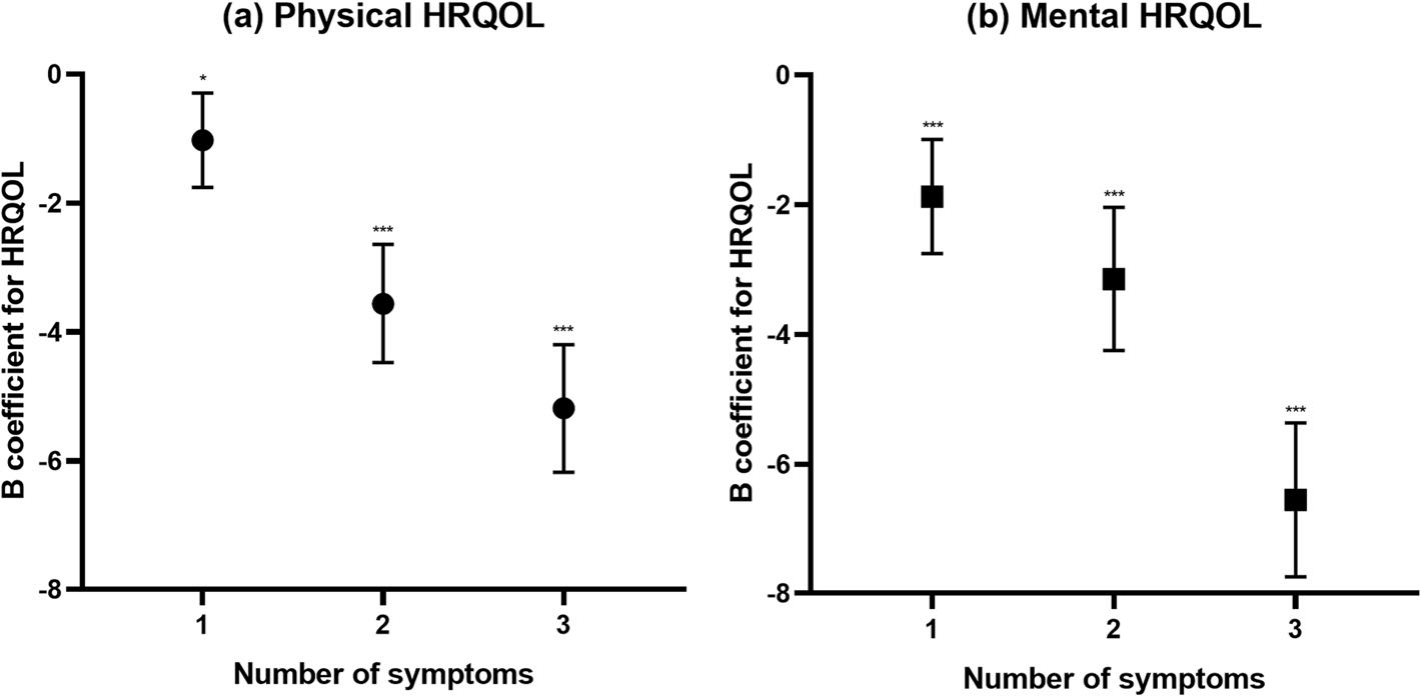
Association between number of symptoms and HRQOL. HRQOL: health-related quality of life. ^*^P < 0.05, ^***^P < 0.001. Linear regression model for physical and mental HRQOL with number of symptoms as a dummy variable (no symptoms as the reference group) after adjusting for sex, age, region, occupation, income, education, marital status, smoking, alcohol consumption, physical activity, and disease history. (**a**) Linear regression of PCS; B = − 1.01, − 3.55, − 5.18 for one, two, and three or more symptoms, respectively. (**b**) Linear regression of MCS; B = − 1.87, − 3.14, − 6.55 for one, two, and three or more symptoms, respectively

Concerning the number of symptoms, 38.6% people had none, 30.9% people had one, 16.0% had two, and 14.5% had three or more. Number of symptoms was associated with both physical and mental aspects of HRQOL after adjusting for all variables. Both the PCS and MCS degraded linearly with an increasing number of symptoms as shown in Fig. 3.

### Associations between individual and combined symptoms and HRQOL

Regarding individual symptoms, fatigue was the most frequently reported symptom (53.7%), followed by pain (24.7%), digestive problems (18.4%), and sleep disturbances (16.3%). All individual symptoms had a significant association with both PCS and MCS in the linear regression analysis. Pain (B = − 5.63) showed the largest effect on PCS, and sleep disorder (B = − 4.43) and digestive problems (B = − 4.18) showed a large degrading influence on MCS.

Concerning combined symptom patterns, fatigue only was the most common (24.8%), followed by fatigue and pain (8.2%), and all four symptoms (6.5%). In the linear regression analysis of the PCS and MCS HRQOL, fatigue only led to a decrease in MCS, but not in PCS; whereas pain only was related to a decrease in PCS, but not in MCS. By contrast, the mix of fatigue and pain was linked to lower PCS and MCS. Three or more symptoms, when combined, showed a similar association with both PCS and MCS. Having all four symptoms was the symptom pattern with the strongest association with HRQOL. (Table 2).

**Table 2:** Influence of reduced HRQOL per individual symptoms and symptom combinations

Symptom	n (%)^**a**^	Physical HRQOL		Mental HRQOL	
		B coefficient^**b**^ (95% CI)	***P-***value	B coefficient^**b**^ (95% CI)	***P-***value
**Individual symptom**					
Fatigue (yes)	591 (53.7)	−2.12 (− 2.76, −1.47)	< 0.001	− 2.95 (−3.72, − 2.19)	< 0.001
Pain (yes)	272 (24.7)	−5.63 (− 6.35, − 4.91)	< 0.001	− 2.86 (− 3.79, − 1.92)	< 0.001
Digestive problems (yes)	202 (18.4)	− 2.02 (− 2.87, − 1.17)	< 0.001	−4.18 (− 5.17, − 3.18)	< 0.001
Sleep disturbances (yes)	179 (16.3)	−2.35 (− 3.24, − 1.46)	< 0.001	− 4.43 (− 5.39, − 3.29)	< 0.001
**Combinations of symptoms**^c^					
None	424 (38.5)	Reference		Reference	
Fatigue only	273 (24.8)	−0.61 (− 1.35, 0.14)	0.109	−1.85 (−2.77, − 0.93)	< 0.001
Pain only	31 (2.8)	−5.96 (− 7.77, − 4.16)	< 0.001	−1.27 (− 3.50, 0.97)	0.266
Fatigue + pain	90 (8.2)	−6.09 (−7.21, −4.97)	< 0.001	− 2.97 (− 4.35, − 1.58)	< 0.001
Fatigue + digestive problems	38 (3.5)	−0.98 (− 2.59, 0.63)	0.231	− 4.16 (− 6.14, − 2.17)	< 0.001
Fatigue + sleep disturbances	35 (3.2)	−0.73 (− 2.43, 0.94)	0.389	−3.34 (− 5.43, − 1.24)	0.002
Fatigue + pain + digestive problems	35 (3.2)	−4.23 (− 5.94, − 2.51)	< 0.001	− 4.43 (− 6.74, − 2.50)	< 0.001
Fatigue + pain + sleep disturbances	29 (2.6)	−6.16 (− 8.00, − 4.33)	< 0.001	− 4.51 (− 6.78, − 2.25)	< 0.001
All four symptoms	72 (6.5)	−6.88 (−8.18, − 5.58)	< 0.001	−7.63 (− 9.24, − 6.02)	< 0.001

*HRQOL* health-related quality of life, *CI* confidence interval. ^a^Percentage (%) is the number (n) divided by 1100 (total N). ^b^Unstandardized B coefficient. ^c^Seven patterns out of 16 symptom patterns were excluded from the analysis (*n* = 73). Linear regression model for physical and mental HRQOL after adjusting for sex, age, region, occupation, income, education, marital status, smoking, alcohol consumption, physical activity, and disease history

## Discussion

We examined the association between four common symptoms—fatigue, pain, digestive problems, and sleep disturbances—and HRQOL in the general population of South Koreans. Overall, one in every two participants lived with discomfort from fatigue, about one-third of them were found to have two or more concurrent symptoms, and symptoms including fatigue and pain were the most common. The influence of reduced physical and mental HRQOL significantly differed for numbers of symptom and type of combined symptoms. These results elucidate why it is important to try to prevent fatigue, pain, digestive problems, and sleep disturbances in the general South Korean population, and how the relationship between those symptoms affects people’s HRQOL. Therefore, we suggest that, when individuals present with key symptoms, they are not dismissed as being simply uncomfortable; rather, these symptoms should be addressed with priority to promote better HRQOL. Furthermore, the results inform approaches and strategies that utilize various methods to manage complex symptoms.

In this study, approximately half of participants complained of fatigue, and about 16–25% complained of pain, digestive problems, and sleep disturbances. The relationships between symptoms and physical and mental HRQOL showed different trends according to individual symptoms after adjusting for sex, age, and the remaining three symptoms not being used as the dependent variable. Fatigue, digestive problems, and sleep disturbances demonstrated a significant association with mental HRQOL, while pain showed a significant association with physical HRQOL. The assessment of HRQOL is a multidimensional concept that covers physical, psychological, and social domains of health [34]. Consequently, we examined both physical and psychological aspects of HRQOL, using the widely adopted SF-12 [29]. While previous research also observed that fatigue, pain, and sleep disorders were associated with all domains of the EQ-5D [35], another representative generic measure of HRQOL, digestive problems were related to only one category of the EQ-5D: anxiety/depression [12]. Although there is some discrepancy between previous research and this study concerning the association between individual symptoms and physical/mental HRQOL, the two are similar in that both found that a specific symptom may affect a specific category of HRQOL.

As the number of symptoms increased, both physical and mental HRQOL showed a significant decrease. Simply put, the number of symptoms was associated with HRQOL. Our results correspond to those of a study that examined the general population in the Netherlands, in which the number of medically unexplained physical symptoms was strongly associated with worse HRQOL as compared to medically explained symptoms [9]. Furthermore, in previous studies, the association between the number of symptoms and HRQOL showed different trends according to age group. No association between the number of symptoms and HRQOL was found in people aged 65 years or older, and there was a weak association in younger or middle-aged people [9, 36]. It is thought that, because elderly people may think of symptoms such as pain and sleep disorders as inevitable issues that come with age, they may cope better or more easily and accept their symptoms, thus attenuating the impact on HRQOL [37]. In this study, age was an adjusted variable; thus, it is necessary to investigate the relationship between the number of symptoms and HRQOL according to age group in future studies.

Interestingly, the symptoms patterns mostly frequently included both fatigue and pain. Roughly one-fifth of participants experienced fatigue and pain, with or without other symptoms. Further, the association between symptoms and physical/mental HRQOL varied depending on the symptoms’ pattern after adjusting for all variables. Specifically, physical HRQOL was strongly associated with comorbid fatigue and pain. This was similar to the score estimated when all four symptoms affecting the highest decrease in physical HRQOL were present. Regarding mental HRQOL, the association was highest when all four symptoms were present. In previous studies, physical symptom clusters that included pain, fatigue, and sleep disorders were observed in patients with multiple sclerosis, and they had a greater influence on HRQOL than did emotional/cognitive or motor symptom clusters [38]. The influence on the outcome also varied depending on the categorization of subgroups according to symptom levels of fatigue, sleep disorders, depression, and pain. Participants in the subgroup experiencing a high level of all four symptoms reported the lowest functional status and poorest HRQOL as compared to their counterparts [21, 39]; thus, our results are similar to the results observed in previous studies.

The strength of this study is that it involved a nationwide survey targeting the general population and excluded bias by stratifying participants according to sex, age, and region. Further, each of the symptoms was defined regarding severity, duration, and degree of recovery. Moreover, the face-to-face interviews were conducted by professional researchers using a systematic method.

Nonetheless, this study had a few limitations. First, we measured participants’ symptoms through self-reporting; therefore, some degree of subjectivity likely exists. However, we tried to minimize the possibility of recall bias by asking participants about the symptoms they experienced within the last 30 days. Second, the tool used in this study established an operational definition for individual symptoms owing to a lack of validated cut-offs. However, the prevalence rates of each symptom in the general population group (fatigue: 17–35.4% [40, 41], pain: 17–20% [42], sleep disorder: 17% [43], digestive problem: 18% [44]) obtained from symptom-specific questionnaires or medical clinics of previous studies were partially similar to the distribution of each symptom in this study; therefore, we believe that the tool used in this study reflects the characteristics of symptoms to a certain extent. Third, this study could not correct for the influence of non-response bias. According to a recent study, the survey response rate was approximately 28% [45]. Nevertheless, we conducted surveys in cooperation with a social research agency and tried to minimize non-sampling error during sampling and data collection steps. Fourth, psychological aspects such as depression and anxiety, and how they relate to the four symptoms frequently encountered in clinical settings, need to be considered in a further study. Lastly, because we employed a cross-sectional design, causality cannot be inferred.

## Conclusions

We investigated the relationships between individuals’ fatigue, pain, digestive problems, and sleep disturbances with their HRQOL. The number and pattern of symptoms varied and displayed distinct associations with physical and mental HRQOL. This highlights that extra attention should be given to symptom patterns, especially in individuals experiencing both fatigue and pain, and individuals with three or more symptoms. The current results provide useful information for caregivers and medical workers who wish to improve patients’ HRQOL and plan treatment for people experiencing a variety of symptoms. Researchers should continue to investigate the relationships between symptoms and HRQOL using more advanced study designs and statistical methods.

## Data Availability

The data supporting the current findings are available from the Korean Medicine Data Center (http://kdc.kiom.re.kr), although they are not freely available. Researchers who fulfill the criteria for access to confidential data can apply for access.
